# Medical and moral considerations regarding complex medical decisions in older patients with multimorbidity: a compact deliberation framework

**DOI:** 10.1186/s12877-018-0707-5

**Published:** 2018-01-25

**Authors:** Jeroen F. A. M. Janssens, Susanne J. de Kort, Wilco P. Achterberg, Susan Kurrle, Ngaire Kerse, Ian D. Cameron, Dorothea P. Touwen

**Affiliations:** 1 Department of public health and primary care, Leiden University Medical Center, Postal adres: V06-P, Postbus 9600, 2300 RC Leiden, The Netherlands; 2 Novicare, Laan van Vredenoord 33, 2289 DA Rijswijk, The Netherlands; 3 Topaz-Zuydtwijck, Aaltje Noordewierlaan 50, 2324 KS Leiden, The Netherlands; 4 Department of public health and primary care, Leiden University Medical Center, The Netherlands, Postal adres: V06-P, Postbus 9600, 2300 RC Leiden, The Netherlands; 5 Curran Ageing Research Unit, University of Sydney, Hornsby Ku-ring-gai Health Service, Hornsby, NSW 2077 Australia; 6 School of Population Health, University of Auckland, Tamaki Campus, Private Bag 92019, Auckland, 1001 New Zealand; 7 John Walsh Centre for Rehabilitation Research, Sydney Medical School Northern, Kolling Institute, Royal North Shore Hospital, St Leonards, NSW 2065 Australia; 8 Department of Medical Ethics and Health Law, Leiden University Medical Centre, The Netherlands, Postal adres: J1-P, Postbus 9600, 2300 RC Leiden, The Netherlands

**Keywords:** Medical decision, Elderly, Multimorbidity, Shared decision making, Intercurrent disease

## Abstract

In health care for older adults, patients with multimorbidity usually receive the same interventions as those patients without multimorbidity. However, standard curative or life-sustaining treatment options have to be considered carefully in view of the maximally attainable result in older and frail patients. To guide such complex medical decisions, we present a compact deliberation framework that could assist physician(s) in charge of the medical treatment of a specific elderly patient to systematize his own thinking about treatment and decisional responsibilities, in case of an intercurrent disease.

The framework includes four questions to be addressed when deciding on a single urgent standard curative or life-sustaining intervention in acute medical problems of an elderly patient with multimorbidity: 1) What is known about the patient’s aims and preferences? 2) Will the intervention be effective? 3) Will the intervention support the aims and preferences of the patient? 4) In view of the aims and preferences, will the risks and benefits be in balance?

If all four considerations are answered favorably, the intervention will fit patient-centered and appropriate care for frail older patients with multimorbidity.

Application to a patient case illustrates how our framework can improve the quality of the shared decision-making process in care for older people and helps clarify medical and moral considerations regarding how to appropriately treat the individual patient.

## Background

In patients with multimorbidity, decision-making as to whether or not to treat a particular affliction is a complex matter, with a need to balance the medical considerations and the patient’s aims and wishes [1–4]. Many treatment decisions concerning frail older patients with multiple health conditions are preference-sensitive. In each new situation a new decision has to be made not only regarding the benefits and risks of the intervention, but also regarding how the intervention fits in with the patient’s wishes. Every physician has to weigh these matters on a regular basis.

We present a compact framework for deliberation by the physician that helps to clarify the core elements of the clinical ‘weighing process’, making explicit the different elements. Developed in nursing home medicine it is especially helpful when faced with an acute treatment decision for a single standard intervention in case of an intercurrent disease in the context of a patient with multimorbidity. The framework helps to distinguish who has the final say in the different elements of deliberation. Although thoroughly incorporating the patient’s perspective, the framework is not meant as a decision aid for physician-patient communication or as an alternative method for shared decision-making. Rather, it helps clarify and articulate the thinking process of the physician when choosing which course of action is advisable, taking into consideration the perspectives of both the patient and physician. The framework helps lay the groundwork for the dialogue between physician and patient, or proxy decision-maker.

The framework is presented and illustrated by analyzing a case from our practice in geriatric medicine.

## Main text

Mrs. W., a 92-year-old widow with three daughters, has a medical history of myocardial infarction, cardiac failure, a total hip arthroplasty for osteoarthritis of her right hip, and dementia (probably Alzheimer’s disease). Mrs. W. was admitted to our nursing home because of self-neglect and refusal of help. She could walk independently, needed help for washing and dressing, but was continent. Mrs. W. resided in our facility for approximately 18 months. During this time, she consistently resisted her stay in the institution, stating repeatedly “This is not my kind of life!”. Each day she repeated that she hoped that this day would be her last one. It was obvious to everyone that Mrs. W. was not happy in her cognitively impaired state; she did not have a clinical depression. Because of her lack of competence to make decisions, one of her daughters was her proxy decision-maker. In earlier discussions between the family and physician it was decided that the primary goals of all medical intervention should be to relieve suffering, improve her comfort, and not to extend her lifespan.

Following an upper respiratory tract infection, Mrs. W. had a bad fall. She indicated severe pain in her right upper leg, which was obviously shortened and internally rotated. Clinically the differential diagnosis was a dislocated total hip arthroplasty, a broken total hip prosthesis, or a fracture distal from the hip prosthesis. The administered pain control seemed to relieve her pain. To obtain a clear view of the treatment options, an X-ray was performed in the nearby hospital; this showed a femoral shaft fracture below the level of the total hip prosthesis.

This case highlights the possible difficulties faced in medical treatment decisions due to multimorbidity and frailty. The main focus was whether or not the patient should undergo surgical fracture repair. To clarify our options, the following questions (which form the compact deliberation framework) were considered (Table 1):

1. What is known about the patient’s aims and preferences?
2. Will the intervention be effective?
3. Will the intervention support the aims and preferences of the patient?
4. In view of the aims and preferences, will the risks and benefits be in balance?

**Table 1:** Compact Deliberation Framework applied to Mrs. W

Question	Answer concerning Mrs. W.	Final say
What is known about the patient’s aims and preferences?	• Urge to walk and to move freely • Desire to lead her own former life, NOT to live in a nursing home • Wish to die (she says daily “I hope I will not wake up tomorrow”) • Quality of life and well-being more important than extending life; therefore important to keep her as comfortable as possible	Mrs. W. and Mrs. W.’s daughter in the function of proxy decision maker
Will the (surgical) intervention be effective?	• Operating hip fracture in a patient with dementia has a bad prognosis [8, 9] • Little chance of operating the sub prothetic fracture successfully so Mrs. W. can walk again • Great chance of complications (blood loss, delirium, incontinence a.o.) • Rehabilitation will be complex because of her dementia [10]	Physician
Will the intervention support the aims and preferences of the patient?	• Little chance she will be able to walk independently • Surgical treatment will not make any difference concerning her having to live in a nursing home • Probably surgical treatment will increase suffering	Mrs. W.’s daughter in the function of proxy decision maker
In view of the aims and preferences, will the risks and benefits be in balance?	• In close contact with her proxy decision maker it was decided that the risks and benefits are not in balance since Mrs. W.’s most important wish to return to her former life as non-demented person, is impossible to achieve • There is a much more effective treatment to relieve suffering, namely palliative care.	Physician together with Mrs. W.’s daughter in the function of proxy decision maker

### What is known about the patient’s aims and preferences?

The first step in our compact deliberation framework is to consider the aims and preferences of the patient. Many treatment decisions concerning frail older patients with multiple health conditions are preference-sensitive [1]. Typically, in chronic aged care the aims and preferences of the patient should be explored over time, as they are highly influential in the planning of care, and in balancing the pros/cons of possible interventions [5]. What do we know about the patient? What contributes to her wellbeing and what causes her suffering? Which circumstances improve or sustain her quality of life and how does this compare to the acute situation that she is currently in? This step in the framework directs our thinking towards this specific patient and how we might best help her. These considerations originate from the considerable value attached to patient-centered care, and address the aims and preferences of the individual patient [1, 6]. In the case of an incompetent patient (e.g., Mrs. W. with severe dementia), an advance directive (living will) may need to be scrutinized, and a proxy decision-maker will play an important role in elucidating what the patient would have wanted and what constitutes the patient’s interests. When the patient has become incompetent her former wishes are important, as is a careful estimation of her actual desires and quality of life [7]. Ideally, the aims and preferences of the patient are known to the treating physician prior to the acute situation; in a chronic care setting this is often the case. However, also when a patient is hitherto unknown to the physician, an important first step is to carefully determine what is known about the recent quality of life of this particular patient, and how to sustain or improve it. Mrs. W. did not have an advance directive. However, we knew from her proxy and from Mrs. W.’s stay in our facility that she was not happy. It was clear, also in her cognitively impaired state, that her wish was not to live in the nursing home and that she longed for the end. Therefore, during the time that she still had, all the care was aimed at keeping Mrs. W. as comfortable as possible.

### Will the intervention be effective?

The second question in our framework is to consider the effectiveness of the intervention. Effectiveness is determined by mortality, life expectancy, morbidity, complications, relief of symptoms, improvement in quality of life and, for the specific patient, the chance of attaining the preferred aim. The physician has to translate the currently available evidence of the intervention to this particular patient. If an intervention is not effective, administration of the therapy will not be beneficial and, therefore, cannot be justified. The physician has the ‘final say’ regarding the effectiveness of the considered intervention. In medicine, although (in)effectiveness is rarely a certainty, it is necessary to determine which chance of beneficial effect would qualify as being effective. Concerning hip fractures, the effectiveness of surgery in patients with dementia (in terms of survival, morbidity, complications and functional outcome) is much less than in patients without dementia [8, 9]. In the case of Mrs. W., the orthopedic surgeon stated that the major operation required carried a considerable risk of complications (e.g. blood loss, a small chance of firmly attaching the new prosthesis to probably osteoporotic bone, and no alternative surgical procedure) and only a minor chance of succeeding in helping the patient to walk again. And if effective, rehabilitation would probably be lengthened by her dementia [10]. There is no consistent evidence that surgery is superior to non-surgical treatment when it comes to alleviating pain and discomfort in patients with a fractured hip [11]. However, there have been no trials of non-surgical treatment for peri-prosthetic femoral fractures to our knowledge. Non-surgical palliative treatment has been presented as a possibility to treat hip fractures in patients with dementia [12]. Moreover, available literature is primarily concerned with surgical intervention for regular fractures, while our patient suffered from a peri-prosthetic fracture, making a favorable outcome even less likely. Taking all these aspects into consideration, the effectiveness of surgical intervention for this particular fracture was not convincing.

### Will the intervention support the aims and preferences of the patient?

The third question combines possible medical outcomes with the aims and preferences of the patient. Treatments aimed at improving one outcome (e.g., survival) may worsen another (e.g., function). Therefore, the combination of the patients’ perspective and the medical (im)possibilities is of decisive importance. Deciding on this aspect is, of course, generally the prerogative of the patient. However, in the case of an incompetent patient, a proxy decision- maker plays an important role in consenting to a course of treatment whilst also wishing to respect the interests of the incompetent patient. To decide whether or not the best possible outcome is consistent with the preferred aims, the patient and/or the proxy require adequate information from the physician concerning the estimated possible effectiveness of the intervention. In accordance with the precept of informed consent, a patient’s refusal drastically limits the possibility of a physician to apply the intervention. Weighing former wishes and the current situation, combined with estimation of the medical possibilities, the proxy may take the place of the patient in the dialogue with the treating physician. In our case, because of the inconclusiveness of the effectiveness of the intervention, the proxy was hesitant. Her opinion was that her mother’s most important aim was *not* to live in a nursing home; however, an alternative was impossible because of her dementia. Because the daughter wanted her mother to be as comfortable as possible, she seriously doubted the benefits of surgery.

### In view of the aims and preferences, will the risks and benefits be in balance?

The fourth element of our framework is balancing the harms and benefits of the intervention in view of the patient’s aims and preferences. Are the probable harms sufficiently compensated by the expected benefits? Is the best possible outcome of the therapy good enough to justify the burden and possible suffering? This aspect is not addressed solely by either the patient or physician, but has to be answered by means of a thorough weighing of medical and patient perspectives. This element of deliberation is not necessarily concerned with financial matters. It primarily focuses on the process of deciding how the interests of the patient can be optimally promoted. In the case of an incompetent patient, the responsibility for balancing the harms and benefits must be shared by physician and proxy, incorporating all that is known about the patient’s preferences.

In the case of Mrs. W., it was decided that on balance (given all the uncertainties concerning effectiveness) an operation would not be justified in view of the substantial risks. Earlier on, the daughter and physician had agreed that the ultimate aim of care was to keep Mrs. W, as comfortable as possible. The aim of the intervention was to relieve suffering, not to extend her lifespan. Even though surgery might help to control pain, in this particular case the physician judged that opioid analgesics would also be effective; this was considered to be a better option than undergoing surgery. All things considered, the physician expected to meet the aims and preferences of the patient by means of adequate palliative care. Therefore, after the X-ray, Mrs. W. returned to the nursing home and was kept comfortable with intensive palliative care, including effective pain control.

## Discussion

Our compact deliberation framework regarding whether or not to advice a certain curative or life-sustaining intervention, has helped us when difficult decisions need to be made for acute medical intervention for patients with multimorbidity and an intercurrent disease. The framework provides guidance on weighing the elements that together constitute the medical and moral justification of the optimally patient-centered and appropriate treatment, thoroughly applying medical knowledge to the particular patient with their individual aims and preferences. The framework helps clarify the different aspects of a medical decision and identifies the various responsibilities in this decision-making process. Also, it secures maximum involvement of the concerns of the individual patient. It is primarily aimed at the deliberation process of the physician, helping to prepare for the shared decision making process with patient and/or family, distinguishing decisional responsibilities.

The framework originated from the debate on whether or not to treat [13], in which the question as to whether or not an intervention is futile is a fundamental part of medicine [5]. Our framework provides the individual clinician with a tool to structure thinking. Although it bears resemblance to the so-called Four Quadrants method [14] it was developed independently. Compared to the Four Quadrants method our framework aims at quick application in daily clinical practice, structuring the physician’s thinking, in a situation when it is impossible to organize a clinical ethics and/or a multidisciplinary meeting. To help making complex decisions there are many methods and decision trees available, sometimes developed for specific local or national circumstances [15]. However, the significant surplus value of our framework is that it offers a convenient and simple way for the individual clinician to thoroughly weigh relevant elements of consideration when confronted with a single and/or acute decision in the context of a patient with multimorbidity. Compared to other methods our framework does not aim to create an extensive overview of all relevant elements; instead it helps the physician to concentrate on the key issues and key responsibilities in this acute situation concerning this patient.

Although in the case of Mrs. W. a decision was made to forego surgical intervention, this is not necessarily the outcome of the framework. It is relatively easy to imagine Mrs. W. in a slightly different situation. For example, if she had not yet had a hip replacement, the chance of the success of surgery and rehabilitation would have been more favorable. Also, had her ability to walk been crucial to relieving her agitation, this might also have favored a surgical intervention.

The framework primarily helps to elucidate the thinking process and to clarify the elements that contribute most to any ultimate decision. It should be noted that, in the weighing of the effectiveness, differences may exist between countries [16]. For example, in some cultures, preserving life at any cost is considered a worthwhile aim. The medicolegal systems and societal influences also differ between countries and this framework extends the debate beyond the intensive care unit [17] potentially providing a framework that may be acceptable across systems. Also, balancing of the harms and benefits may turn out differently if, for example, the patient lives in a nursing home or in the community [18], in a rural area and has to travel a considerable distance to receive treatment, or if appropriate postoperative support is not available. Therefore, both the practical and clinical feasibility of the treatment are important factors to consider [1]. Another consideration may be whether a patient is able to (co)pay for the intervention, or whether treatment is covered by the patient’s insurance. The legal circumstances may differ in different countries, giving physicians more or less room to reach their own decision. For example, if the patient’s wish overrules the medical assessment of effectiveness, the framework will help make explicit the arguments that lead to the decision to treat, including those legal restraints, thereby improving the transparency of the medical decision. Considering the fact that patient and family are often not familiar with complex medical weighing processes, the framework will help the physician to organize his thinking, thus facilitating the counseling needed in order to reach an optimal decision that is acceptable to all parties involved. Especially the distinction between responsibilities may help to clarify matters when cultural or religious considerations influence the view of patient or family on what should be done. However, all these cultural and national differences may be taken into account when applying the framework, resulting in a carefully balanced outcome. The framework will help to make explicit all these factors and influences.

## Conclusion

Medical decisions in geriatric care have to incorporate many different elements in order to reach the best decision for elderly patients with multimorbidity and an intercurrent disease. Use of the presented compact deliberation framework may help to achieve a good balance between effectiveness, probabilities, burdens, harms, aims and preferences, making various considerations more explicit and clearly delineating the ‘final say’ of the parties concerned. Hopefully the framework may serve as a tool for physicians involved in the care of older adults, helping them improve their decision-making in (semi) acute situations, and providing a transparent process with a central position for the patient’s preferences.
